# Honor Walk – ein respektvoller letzter Weg rund um die Organspende

**DOI:** 10.1007/s00063-025-01344-y

**Published:** 2025-11-17

**Authors:** Daniela Jacobs, Carsten Hermes

**Affiliations:** 1 Klinik und Poliklinik für Neurochirurgie, Universitätsklinikum Bonn, Venusberg-Campus 1, 53127 Bonn, Deutschland; 2 Hochschule für Angewandte Wissenschaften Hamburg (HAW Hamburg), Hamburg, Deutschland; 3 Akkon-Hochschule für Humanwissenschaften, Berlin, Deutschland

Die Betreuung von Organspendern endet nicht mit der Diagnose des irreversiblen Hirnfunktionsausfalls (IHA). Am Universitätsklinikum Bonn (UKB) wurde ein pflegerisch initiierter, strukturierter Prozess etabliert, der Angehörigen Raum für Trauer gibt, die Würde des Spenders wahrt und Teammitgliedern Reflexion ermöglicht. Der Honor Walk steht dabei exemplarisch für die ethischen, emotionalen und organisatorischen Dimensionen.

## Ausgangspunkt

Angehörige von Organspendern erleben die Phase zwischen IHA-Diagnose und Organentnahme häufig als entfremdend, distanziert und leidvoll. Eine qualitative Studie von Latifi et al. (2025) bestätigt: Familien wünschen sich bessere Beratung, mehr kulturelle Sensibilität beim Thema Hirntod und transparente Kommunikation über den Spendeprozess und die Organvergabe [1].

## Definition und Grundlagen

Honor Walks – stille Zeremonien – begleiten den Übergang eines Menschen zum Organspender. Angehörige und Mitglieder des Behandlungsteams bilden ein Spalier auf dem Weg von der Intensivstation, das Raum für Anteilnahme und Innehalten im Klinikalltag schafft.

Entstanden ist dieses Ritual in den USA. Die Koordination umfasst mehrere Schritte, von der Aufklärung der Familie über die Darstellung der Möglichkeiten bis hin zur Organisation des Krankenhauspersonals und der Kommunikation mit den entsprechenden Abteilungen.

## Honor Walks als Brücke zwischen Leben und Tod

Honor Walks sind mehr als eine Geste. Sie markieren den Übergang eines Patienten in die Rolle einer Person, die Leben schenkt. Ronald Grimes Analysen zeigen: Die Inszenierung dient weniger der individuellen Ehrung, sondern vielmehr der öffentlichen Aufwertung der Organspende an sich. Die Zeremonie spendet Trost und Sinn und normalisiert zugleich einen emotional und ethisch sensiblen medizinischen Prozess, was eine bewusste Reflexion erforderlich macht [2, 3]. Die Integration in Protokolle zeigt deren wachsende Bedeutung in der Transplantationsmedizin [4].

Das California Transplant Donor Network evaluierte diesbezüglich ein spezifisches Abschiedsritual. Familien konnten eine persönliche individuelle „Ehrenerklärung“ formulieren (frei formulierter, kurzer Text, der dem verstorbenen Spender öffentlich Respekt und Dankbarkeit ausdrückt), die im OP-Saal vorgelesen wurde, gefolgt von einem Moment der Stille im OP vor der Organentnahme. Dieses ritualisierte Vorgehen wurde 2011 in 22 Kliniken in der USA erprobt und insgesamt 71-mal umgesetzt. Die Rückmeldungen der Familien bestätigten, dass sie sich durch das Ritual in ihrem Wunsch, den Verstorbenen zu ehren, unterstützt fühlten [5].

Auch während der COVID-19-Pandemie gelang es einer Klinik, einen Honor Walk umzusetzen. Eine eindrucksvolle Ausnahme in einer Zeit, in der die bewährte Praxis, den Flur zu säumen, vielerorts pausiert werden musste [6].

## Implementierung am Universitätsklinikum Bonn

Die erfolgreiche Implementierung eines ritualisierten Umgangs mit Organspendern auf der interdisziplinären Neurointensivstation (NICU) am UKB erforderte eine sorgfältige organisatorische Planung und Koordination zwischen verschiedenen Stakeholdern.

**Abb. 1:**
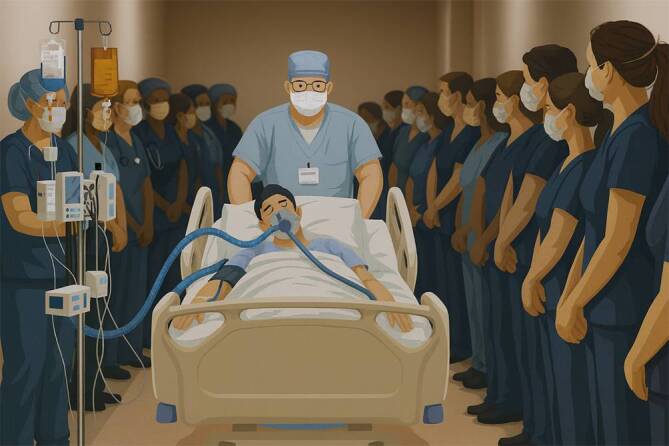
Illustration eines Honor Walk (erstellt mit ChatGPT 5.0)

Wichtig: Die Teilnahme am Honor Walk (Abb. 1) ist freiwillig und führt zu keiner bevorzugten Patientenbehandlung. Seelsorge, Kriseninterventionsteam (KIT), Deutsche Stiftung Organtransplantation (DSO) sowie die Stationsleitungen stehen jederzeit als Gesprächspartner für alle Beteiligten auch ohne eine Teilnahme am Honor Walk zur Verfügung.

Die Einführung stieß im klinischen Alltag zunächst auf spürbare Vorbehalte. Geäußert wurden zeitliche Bedenken, Fragen zur Zuständigkeit sowie Zweifel an der Wirksamkeit. Dabei wurde deutlich, dass viele Teammitglieder ihre persönliche Haltung zur Organspende noch nicht reflektiert hatten und die emotionale Dimension, einschließlich Unsicherheiten oder Ängsten, als belastend erlebten. Skepsis riefen insbesondere die medialen Darstellungen aus anderen Ländern hervor, die als potenziell martialisch wahrgenommen wurden.

Hierzu wurde ein interprofessioneller Lösungsansatz im klinischen Alltag der NICU etabliert. Bereits in der Frühphase potenzieller IHA-Verläufe, etwa bei schwerwiegenden neurologischen Ereignissen, werden Transplantationsbeauftragte aktiv eingebunden. Sie nehmen täglich an den multiprofessionellen Frühbesprechungen teil, die ärztliche, pflegerische und therapeutische Berufsgruppen zusammenführen. Dadurch verfügen sie über einen strukturierten Überblick über potenzielle Spenderfälle und können frühzeitig die Koordination mit der DSO und anderen Beteiligten einleiten. Zusätzlich stehen rund um die Uhr ein psychosoziales Kriseninterventionsteam sowie die Klinikseelsorge zur Verfügung. Diese Unterstützungsangebote werden je nach Bedarf durch das Stationsteam aktiviert. Die Kombination aus struktureller Einbindung, multiprofessioneller Kommunikation und psychosozialer Begleitung ermöglicht tragfähige Prozesse für einen würdevollen Abschied – für Angehörige wie für Teammitglieder.

## Honor Walk – ein Ritual der sichtbaren Wertschätzung

Mit dem IHA beginnt ein sensibler Abschnitt, der nicht nur medizinisch und pflegerisch, sondern auch zwischenmenschlich und kulturell ist. Der Honor Walk findet in der Regel zwischen Nacht- und Frühdienst statt. Mitarbeitende aus Pflege, ärztlichem Dienst, Reinigung, Rettungsdienst, der DSO und den Transplantationsbeauftragten, sowie Angehörige stehen Spalier. Diese stille, achtsame Geste unterbricht den routinierten Alltag, bringt Wertschätzung und Respekt sichtbar zum Ausdruck.

Begleitend zum medizinischen Ablauf wird ein strukturierter Abschiedsprozess gestaltet, der auch emotionale, zwischenmenschliche und kulturelle Aspekte berücksichtigt. Die Patientinnen und Patienten werden, wenn möglich, in Einzel- oder Zweibettzimmer verlegt. Angehörigen wird angeboten, den Raum persönlich zu gestalten. Dies kann beispielsweise durch das Aufhängen von Fotos oder durch vertraute Gegenstände wie einen leuchtenden Stern geschehen, der zu Hause Schutz vor Albträumen symbolisierte.

**Abb. 2:**
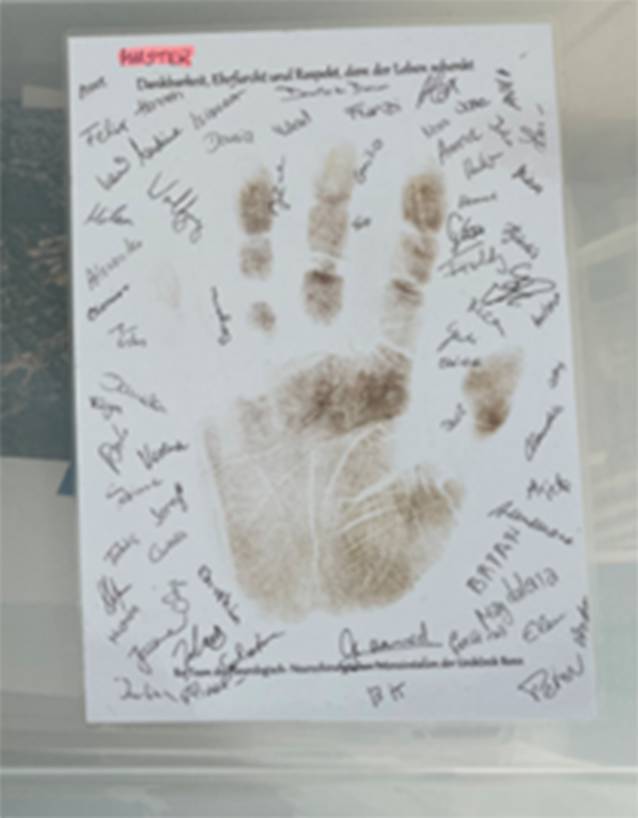
Handabdruck

**Abb. 3:**
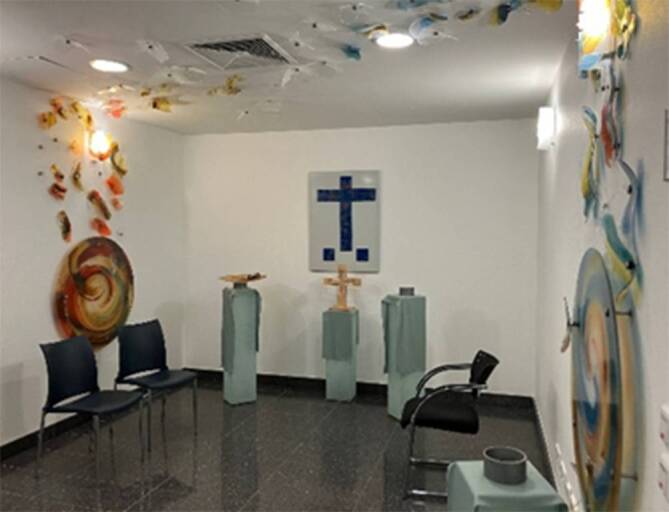
Abschiedsraum

Ein zentrales Element ist die sogenannte Begleitmappe, die individuell vorbereitet wird. Inspiriert von Ritualen der Geburtshilfe, wie dem symbolischen Fußabdruck von Neugeborenen, wird ein Handabdruck (Abb. 2) der Spendenden angefertigt und auf Karton aufgebracht. Alle beteiligten Teammitglieder unterzeichnen diesen Abdruck, als Zeichen von Mitgefühl, professioneller Verantwortung und persönlicher Anteilnahme. Die Mappe enthält zudem einen Umschlag für eine Haarsträhne, die Visitenkarte der Station sowie ein Bild der Krankenhauskapelle als Ort der Erinnerung (Abb. 3, Abschiedsraum). Damit die Umsetzung auch bei hoher Arbeitsbelastung gelingt, stehen vorbereitete Materialsets jederzeit zur Verfügung.

Angehörigen wird angeboten, dass Spendende nach der Organentnahme in ihrer Lieblingskleidung eingekleidet werden, z. B. erhielt eine Spenderin, eine angehende Medizinerin, in Absprache mit der Mutter symbolisch einen Kasack der schichtleitenden Pflegefachperson.

Wann immer möglich, wird der Spendende durch ein Stationsteammitglied in den OP begleitet. Die Angehörigen des Spendenden dürfen der Einschleusung in den OP beiwohnen. Es folgt eine formelle Vorstellung nebst Schweigeminute. Nach der Explantation wird der Körper achtsam versorgt und neu eingekleidet. Im eigens gestalteten Aufbahrungsraum erhalten Angehörige Gelegenheit zum letzten Abschied. Transplantationsbeauftragte und die DSO informieren im Nachgang. Diese Rückmeldungen geben dem multiprofessionellen Team eine spürbare Rückkopplung zur Selbstwirkung.

## Zusammenfassung

Trotz der geringen Anzahl an Organspenden am UKB hat sich der Prozess als tragfähige Struktur erwiesen. Durch die ritualisierte Gestaltung entstand ein Handlungspfad, der nicht nur Sicherheit im Ablauf schafft, sondern auch emotionale Entlastung bietet. Niemand steht mehr unvorbereitet und allein in der Begleitung eines Organspenders, stattdessen wird Zugehörigkeit und gemeinsame Verantwortung erfahrbar.

Zentrale Werte wie Respekt, Dankbarkeit und Menschenwürde werden im Alltag sichtbar gelebt. Die Angehörigen erfahren Raum, Zeit und Geborgenheit für den Abschied. Das Behandlungsteam erlebt durch Rückmeldungen (Briefe der Angehörigen) und persönliche Gesten (Schenkung eines eigenen Sterns) eine tiefe Sinnstiftung. Solche Erfahrungen fördern nicht nur individuelle Reflexion, sondern stärken auch das professionelle Selbstverständnis. Im Team hat sich die Kommunikation zur Thematik Organspende deutlich intensiviert.

Zur nachhaltigen Verankerung wurde ein umfassend geschultes Multiplikatorenteam gebildet. Dessen Mitglieder stehen insbesondere zur Unterstützung jüngerer Teammitglieder sichtbar gelistet als Ansprechpersonen bereit. Ergänzend wird vertieftes Informationsmaterial bereitgestellt, das sowohl zur Angehörigenberatung als auch zur internen Weiterbildung genutzt wird. Die regelmäßige Schulung des Teams gewährleistet eine kontinuierliche Sensibilisierung und qualitative Weiterentwicklung des Gesamtprozesses.

## Fazit für die Praxis

- Strukturierte Prozesse schaffen Sicherheit im Umgang mit Organspenden.
- Ein interprofessioneller Honor Walk zeigt gemeinsamen Respekt für die Organspenderituale, unterstützt Angehörige und fördert das Teambuilding.
- Ein Multiplikatorenteam sichert nachhaltige Umsetzung.

Gerade im technisch geprägten Umfeld der Intensivmedizin zeigt der Honor Walk: „Menschlichkeit ist kein Widerspruch zur Hochleistungsmedizin“.

Einen Eindruck von der Zeremonie erhält man über die folgenden öffentlich zugänglichen Links:

https://www.youtube.com/watch?v=mlMh75eqFPA

https://www.youtube.com/watch?v=WSUx-ZqapBs
